# Supplementary material for: Social information decreases giving in late-stage fundraising campaigns
Source: PLoS One. 2022 Dec 1;17(12):e0278391. doi: 10.1371/journal.pone.0278391 (PMC9714697; doi:10.1371/journal.pone.0278391)
Supplement: S1 Appendix — (DOCX) [file pone.0278391.s001.docx]

# Supporting information

**S1 Appendix**

**Study 1 – JGive.com**

**Website description***.* JGive is a privately held Israeli-based non-profit organization that features over 750 charities to reach potential donors through their digital platform. Each month, JGive processes tens of thousands of donations from individuals across the globe. As with most digital fundraising platforms, potential donors are presented with up-to-date information about the fundraising campaigns which includes the goal progress and the number of donors to the campaigns (see S1 Fig below for example screenshot).

| 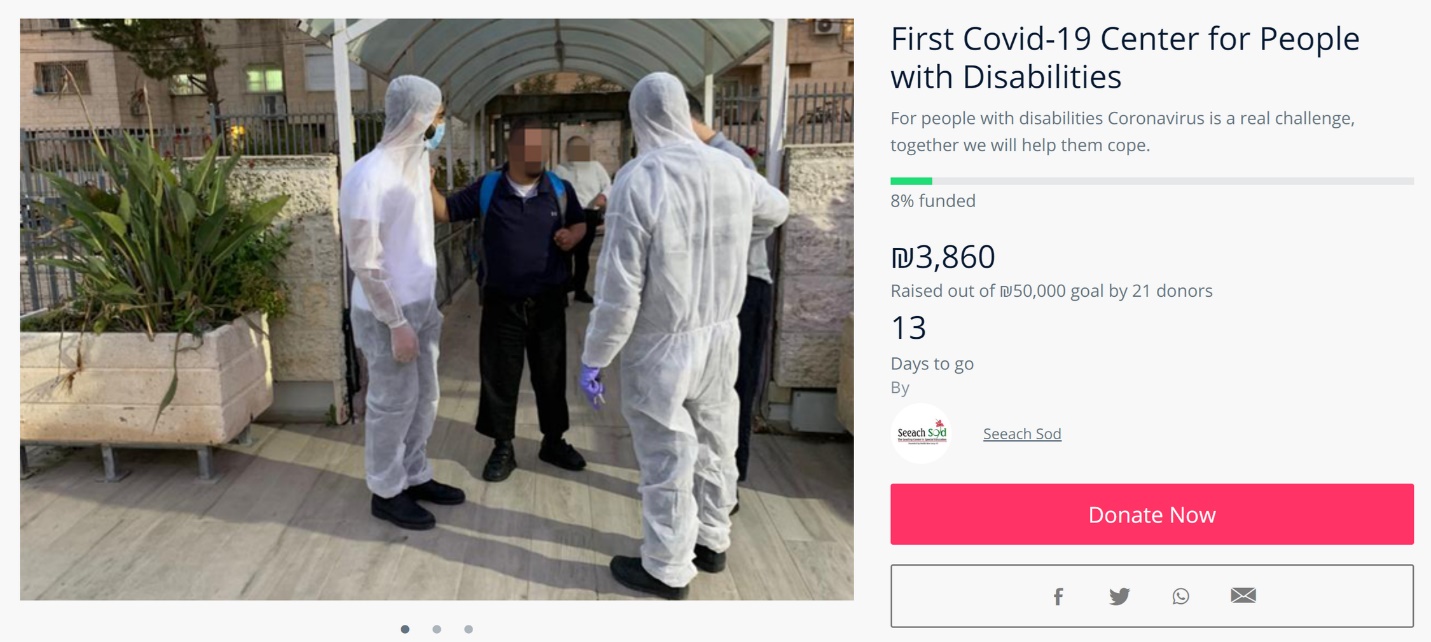 |
| --- |
|  |

*S1 Fig.* Example of JGive Fundraising Page.

**Dataset and sampling procedure**. We collaborated with the JGive.com research department who provided us with the dataset containing information on individual donations on their website. The original dataset included 72,538 donations made on JGive website from November 1^st^ 2019 to March 22^nd^ 2020. Given the spread of the Coronavirus and the unprecedented global crisis that also began to escalate in Israel at the end of February 2020 (dates of first known case and first death in Israel are 2-22-2020 and 3-21-2020, respectively), we suspected that because of the economic downturn as well as a potentially dramatic change in people’s philanthropic decision-making (e.g., putting a greater focus on perceived need), the JGive data amidst the crisis would no longer represent a valid field for testing our hypothesis. While no rigorous investigation that explores donation decisions during the coronavirus crisis had been published yet (2021), the popular press details the challenges the pandemic poses for nonprofit organizations and report significant decreases in the donations they receive.^^[[1]](#footnote-1)^^ Therefore, we excluded data from the month of March 2020. As mentioned in the main text, we also excluded donations that were made after the goal had been reached, as well as those donations that were part of a recurring donation program because these donation decisions are unlikely to be influenced by the investigated factors. Our final sample consisted of 42,702 donations representing 3,147 unique campaigns that were created by 143 charity organizations.

**Study 2**

**Methods**

**Stimuli**. Stimuli for Study 2 is reprinted below. Note that the text in bold was changed based on condition.

We would like to tell you about an organization called Action Against Hunger, a charity that saves the lives of malnourished children, while providing communities with access to safe water and sustainable solutions to hunger.

Action Against Hunger saves lives while building long-term strategies for self-suﬃciency:

- Lifesaving impact in 45+ countries
- Field staﬀ assisting more than nine million people each year
- 35 years of expertise in contexts of conflict, disasters and food crises

Action Against Hunger save hundreds of thousands of lives each year, but millions of malnourished children remain in need of lifesaving treatment.

See S2 Fig below for screenshots of stimuli used in the experiment.
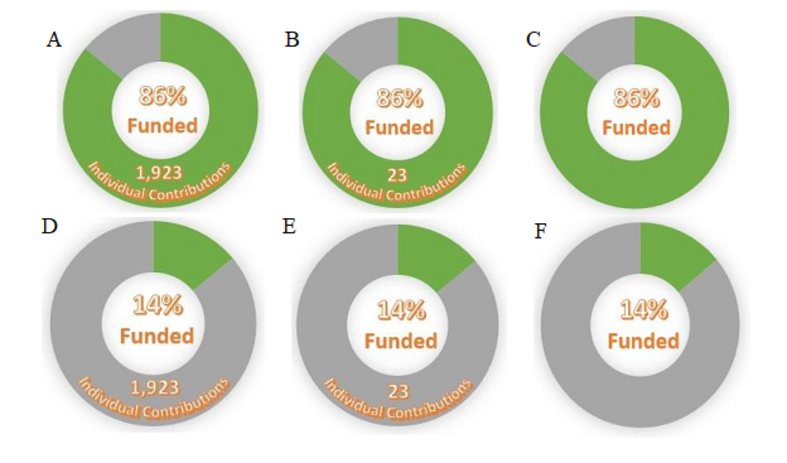


|  |
| --- |
| *S2 Fig*. Screenshots of the experimental conditions, Study 2. Panel A: Close proximity, many donors; Panel B: Close proximity, few donors; Panel C: Close proximity, no social information; Panel D: Far proximity, many donors; Panel E: Far proximity, few donors; Panel F: Far proximity, no social information. |

We are helping Action Against Hunger to raise money for a campaign to fight high rates of chronic child malnutrition in Nicaragua.

**As of now, we have received 1,923 donations for this campaign, and have reached 86% of the campaign goal.**

To help Action Against Hunger raise money for this campaign, we are entering participants of this survey into a lottery. One participant will be randomly selected to receive a bonus of up to $20.

We are oﬀering the chance to donate all, some, or none of that $20 bonus to Action Against Hunger for its life-saving eﬀorts in Nicaragua. If you are selected to win, we will donate any amount you choose to donate to Action Against Hunger, and we will pay you the remaining amount as a bonus.

Your donation will help provide food, water, and other essential items to malnourished children in Nicaragua.

If you are selected to receive the $20 bonus today, how much of the $20, if any, would you like to donate to the charity, Action Against Hunger?

0 5 10 15 20

I would like to

donate ($):

**Follow-up exploratory questions**. As mentioned in the main text, after participants made their donation decision we asked several exploratory follow-up questions.

First, participants were asked a question about perceived progress (adapted from Cryder, Loewenstein, & Seltman, 2013) and the likelihood of the campaign goal being completed (order randomized): “How much of an impact do you think your donation decision will have on reaching the campaign goal?” (1 = *none at all*, 7 = *a great deal*) and “How likely do you think it is that the charity will reach the campaign goal?” (1 = *very unlikely*, 7 = *very likely*).

On the following survey page, participants were asked two more questions concerning perceived progress (Cryder et al., 2013). Specifically, participants were asked: “How much progress would your potential donation make toward the campaign goal?” and “How substantial would your contribution be toward the campaign’s goal if you decided to donate?” We also asked two questions to measure participants’ anticipated positive affect towards giving: “How happy would you feel to help the charity reach its campaign goal?” and “How satisfying would it be to help the charity to reach its campaign goal?” All questions were on a 1 (*not at all/none at all*) to 7 (*a great deal/very much*) scale and were displayed in a randomized order.

In addition to exploratory follow-up exploratory questions, we included manipulation check questions to confirm that 1,923 was perceived as a greater number of donors than 23 and that 86% was seen as closer to the campaign goal than 14%. Following these manipulation checks, participants were asked two questions about the importance of the charity’s cause to themselves and to others: “How much do you personally care about the charity’s cause?” and, “How much do you think others care about the charity’s cause?” (1 = *not at all*, 7 = *very much*). They were also asked a question assessing anticipated negative affect elicited by not giving: “How bad would you feel if you did not help the charity?” (1 = *not at all*, 7 = *very much*). Finally, participants reported how frequently they donate to charities (1 = *never*, 2 = *rarely*, 3 = *sometimes*, 4 = *often*, 5 = *All the time*) and whether they have heard of Action Against Hunger before taking the survey (1 = *yes*, 2 = *no*, 3 = *not sure*). The survey concluded with demographic questions.

**Additional Results**

**Charitable giving**. In addition to the two-way ANOVA reported in the main text, we ran two additional models to test the robustness of our main results. In the first model, we added gender and age as control variables and in the second model we log-transformed the donation amount to account for non-normal distribution of standardized residuals (Shapiro–Wilk’s W = .88, *p* < .05). We report these results in S1 and S2 Tables, respectively. As seen in the tables, the significant interaction between number of donors and goal proximity remained robust to inclusion of these covariates.

*S1 Table:* Study 2 results controlling for gender and age. Dependent variable = amount donated.

|  | **Df** | **Sum Sq** | **Mean Sq** | **F value** | **p value** |
| --- | --- | --- | --- | --- | --- |
| Goal proximity | 1 | 3.53 | 3.53 | .06 | .800 |
| Number of donors | 2 | 42.50 | 21.25 | .39 | .680 |
| Gender | 1 | 515.62 | 515.62 | 9.38 | .002 |
| Age | 1 | 14.14 | 14.14 | .26 | .612 |
| Goal prox. X Number of donors | 2 | 430.05 | 215.03 | 3.91 | .021 |
| Residuals | 563 | 30941.54 | 54.96 |  |  |

*S2 Table:* Study 2 results with log-transformed donation amount. Dependent variable = log(amount donated).

|  | **Df** | **Sum Sq** | **Mean Sq** | **F value** | **p value** |
| --- | --- | --- | --- | --- | --- |
| Goal proximity | 1 | 15.31 | 15.31 | .98 | .323 |
| Donors | 2 | 6.21 | 3.11 | .20 | .820 |
| Gender | 1 | 275.86 | 275.86 | 17.62 | .000 |
| Age | 1 | 69.93 | 69.93 | 4.47 | .035 |
| Goal prox. X donors | 2 | 117.56 | 58.78 | 3.75 | .024 |
| Residuals | 563 | 8814.25 | 15.66 |  |  |

**Exploratory mechanism items**. Given the relatively large number of potentially correlated follow-up items measured, we first conducted exploratory factor analyses (maximum likelihood factoring, varimax rotation) on these items. Three factors emerged, accounting for 61% of the variance. All exploratory items’ factor loadings and eigenvalues are provided in S3 Table.

*S3 Table*: Eigenvalues and factor loadings for factor analysis in Study 2. Bold loadings within each factor indicate items that comprised that factor in mediation analyses.

|  | **Factor 1**  *Eigen value = 2.210* | **Factor 2**  *Eigen value = 2.111* | **Factor 3**  *Eigen value = 1.147* |
| --- | --- | --- | --- |
|  |  |  |  |
| 1. How much of an impact do you think your donation decision will have on reaching the campaign goal? | .28 | **.65** | .21 |
| 2. How likely do you think it is that the charity will reach the campaign goal? | .18 | .25 | .39 |
| 3. How satisfying would it be to help the charity reach its campaign goal? | **.84** | .30 | .10 |
| 4. How happy would you feel to help the charity reach its campaign goal? | **.85** | .27 | .16 |
| 5. How much progress would your potential donation make toward the campaign goal? | .24 | **.85** | .15 |
| 6. How substantial would your contribution be toward the campaign’s goal if you decided to donate? | .30 | **.79** | .15 |
| 7. How much do you personally care about the charity's cause? | .54 | .23 | .51 |
| 8. How much do you think others care about the charity's cause? | .08 | .08 | **.76** |
| 9. How bad would you feel if you did not help the charity? | **-.47** | -.24 | -.20 |

We labeled the three constructs identified in the factor analysis as (1) *anticipated emotion* (or warm glow), which is captured by the following items: “How satisfying would it be to help the charity reach its campaign goal?”; “How happy would you feel to help the charity reach its campaign goal?”; and “How bad would you feel if you did not help the charity?” (reverse-scored); (2) *perceived progress*, which is captured by the following items: “How much of an impact do you think your donation decision will have on reaching the campaign goal?”; “How much progress would your potential donation make toward the campaign goal?”; and “How substantial would your contribution be toward the campaign’s goal if you decided to donate?”; (3) *perceived worthiness* *of the cause to others*, as measured by the item, “How much do you think others care about the charity’s cause?” Items measuring the *perceived* *worthiness of the cause to oneself* (“How much do you personally care about the charity’s cause?”) and *likelihood* *of reaching the goal* (“How likely do you think it is that the charity will reach the campaign goal?”) did not load cleanly on any factor and so were treated independently in the analysis. We therefore used five variables as potential mechanisms in mediation analyses, three of which were composite measures: perceived progress (α = .87), anticipated affect (α = .81), perceived worthiness of the cause to others, perceived worthiness of the cause to oneself, and likelihood of reaching the goal.

We ran moderated mediation models using the bootstrapping method with bias-corrected confidence estimates and 5,000 resamples (PROCESS Macro for SPSS, Model 8, Hayes, 2017). Number of donors was entered as the independent variable (1 = few, 0 = many), amount donated as the dependent variable, and goal proximity as the moderator. We examined only those participants in the few donors and many donors conditions, excluding those in the no information control condition.

First, we ran a moderated mediation model containing only our hypothesized mechanism—perceived progress—as a potential mediator. While there was no evidence of moderated mediation (confidence intervals for the index of moderated mediation contained zero), there was a significant indirect effect of perceived progress when close to the goal, β = 0.77, 95% CI [0.03, 1.63]. Those in the few donors condition perceived that their donation would make greater progress towards the campaign goal compared to those in the many donors condition, β = 0.41, *p* < .05. This greater perceived progress in turn translated to higher donations, β = 1.74, *p* < .001. There was no significant indirect effect of perceived progress when far from the goal (confidence intervals contained zero).

We note that neither anticipated affect, perceived worthiness of the cause to oneself or others, nor perceived likelihood of reaching the goal were predicted by the number of donors manipulation (*p*s > .25), thus precluding these variables as mediators of the relationship between social information and giving when close to the goal. Nevertheless, we ran an additional mediation models that included all potential mediators (perceived progress, anticipated affect, perceived worthiness of the cause to others, perceived worthiness of the cause to oneself, likelihood of reaching the goal) entered in the model simultaneously. This model revealed a significant moderated mediation index for anticipated emotion (Index = 1.49, 95% CI [0.08, 3.07]). However, confidence intervals for the indirect effect of this measure at each level of the moderator contained zero. No other potential mediators had a significant moderated mediation index.

Furthermore, we ran the models reported above using the probability of giving as the dependent variable, in lieu of amount donated. With all mechanisms in the model, there was a significant moderated mediation for anticipated affect (Index = 0.63, 95% CI [0.05, 1.43]). However, the indirect effect of anticipated affect was not significant at either level of the moderator. There were no other significant indices of moderated mediation or indirect effects in either model.

These results provide initial evidence that perceived progress underlies the relationship between number of donors and giving when close to the goal. Although there were no significant indirect effects or moderated mediation when all potential mechanisms were included in the model, there was a significant indirect effect of perceived progress in a model with progress entered individually as a mediator. These results, coupled with the finding that neither anticipated affect, perceived worthiness of the cause to others, perceived worthiness of the cause to oneself, nor likelihood of reaching the goal were significantly related to the number of donors manipulation, raise the concern that this experiment was insufficiently powered to detect the indirect effect of perceived progress. However, we note that in Study 3, we replicate Study 2 using a larger sample size, show that perceived progress does indeed mediate this relationship when close to the goal, and that the indirect effect of perceived progress holds in a model containing other potential mediators (see main text).

**Study 3**

**Methods**

**Stimuli**. The stimuli for the accumulated goal progress conditions were identical to the stimuli in the close conditions in Study 2 (see S3 Fig, Panels A and B). The text in additional two remaining goal progress conditions read as follows:

“As of now, we have received 23 [1,923] donations for this campaign, and have only 14% of the campaign goal remaining to be funded.”

The circle graphs for the remaining few and many condition are displayed in S3 Fig.

| 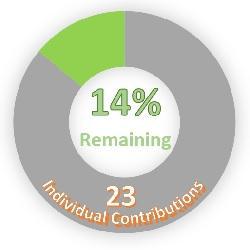A | 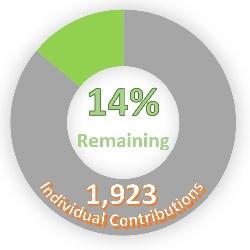 B |
| --- | --- |
|  | |

*S3 Fig*. Screenshots of the remaining conditions, Study 3. Panel A: Remaining goal progress, few donors; Panel B: Remaining goal progress, many donors

**Additional Results**

As mentioned in the main text and illustrated in S4 Fig below, there was no effect of the progress focus manipulation (accumulated vs. remaining) and no interaction.

S4 Fig below displays the results of Study 3.

|  |
| --- |
|  |

**Exploratory measures**. As mentioned in the main text, we measured beliefs about the extent to which the number of donors influenced donation decisions. A two-way ANOVA with number of donors, progress focus, and their interaction entered as factors revealed no main effects and no interactions (*p*s > .25) on this item. Furthermore, while beliefs about the influence of donors was unrelated to the amount given (*p* > .25), it was significantly related to the probability of giving. A greater belief that the number of donors influenced giving decisions was associated with a higher probability of donating, *Coef.* = 0.25, *p* < .001.

**Mediation**. As described in the manuscript, we ran multiple mediation models with number of donors entered as the independent variable (few = 1, many = 0), amount donated as the dependent variable, and the following measures as potential mediators: perceived progress; perceived average donation; importance placed on campaign; perceived importance others place on campaign; anticipated positive affect; anticipated negative affect; likelihood of reaching the goal without one’s donation; perceived need; and moral responsibility. Mediation results are displayed in S4 Table. In addition, we ran the same model except with probability of donating as the dependent variable; these results are shown in S5 Table. As indicated in these tables, there was a significant indirect effect of perceived progress in both models (amount donated as DV: β = 0.21, 95% CI [0.04, 0.46]; probability of donating as DV: β = 0.22, 95% CI [0.04, 0.54]). There were no other significant indirect effects.

*S4 Table*. Mediation results, Study 3. Dependent variable = amount donated.

| **Potential Mechanism** | **Coef.** | **SE** | **95% CI** |
| --- | --- | --- | --- |
| 1. Perceived progress | **0.21** | **0.10** | **0.04, 0.46** |
| 2. Perceived average donation | 0.04 | 0.08 | -0.08, 0.22 |
| 3. Importance placed on campaign | 0.02 | 0.11 | -0.21, 0.25 |
| 4. Perceived importance others place on campaign | -0.04 | 0.05 | -0.17, 0.03 |
| 5. Anticipated positive affect | 0.01 | 0.04 | -0.06, 0.11 |
| 6. Anticipated negative affect | -0.01 | 0.03 | -0.08, 0.04 |
| 7. Likelihood of reaching goal without one’s donation | 0.00 | 0.03 | -0.05, 0.08 |
| 8. Perceived need | 0.04 | 0.06 | -0.07, 0.20 |
| 9. Moral responsibility | 0.13 | 0.12 | -0.08, 0.38 |

*Note*: Coef. = coefficient; SE = standard error; 95% CI = 95% confidence interval. Bold font indicates indirect effects with 95% confidence intervals that do not contain zero.

*S5 Table.* Mediation results, Study 3. Dependent variable = Probability of donating.

| **Potential Mechanism** | **Coef.** | **SE** | **95% CI** |
| --- | --- | --- | --- |
| 1. Perceived progress | **0.22** | **0.13** | **0.04, 0.54** |
| 2. Perceived average donation | 0.01 | 0.06 | -0.09, 0.14 |
| 3. Importance placed on campaign | 0.00 | 0.03 | -0.05, 0.06 |
| 4. Perceived importance others place on campaign | -0.01 | 0.02 | -0.06, 0.02 |
| 5. Anticipated positive affect | 0.01 | 0.04 | -0.07, 0.10 |
| 6. Anticipated negative affect | 0.01 | 0.03 | -0.03, 0.09 |
| 7. Likelihood of reaching goal without one’s donation | 0.00 | 0.01 | -0.03, 0.03 |
| 8. Perceived need | 0.00 | 0.02 | -0.03, 0.04 |
| 9. Moral responsibility | 0.03 | 0.03 | -0.02, 0.11 |

*Note*: Coef. = coefficient; SE = standard error; 95% CI = 95% confidence interval. Bold font indicates indirect effects with 95% confidence intervals that do not contain zero.

1. <https://www.campaignlive.com/article/crippling-impact-coronavirus-nonprofits/1678762> [↑](#footnote-ref-1)
