# Supplementary material for: Social information decreases giving in late-stage fundraising campaigns
Source: PLoS One. 2022 Dec 1;17(12):e0278391. doi: 10.1371/journal.pone.0278391 (PMC9714697; doi:10.1371/journal.pone.0278391)
Supplement: Attachment — Submitted filename: Giving Plos One Response Letter R1.pdf [file pone.0278391.s002.pdf]

PONE-D-22-05379
Social Information Decreases Giving in Late-stage Fundraising Campaigns
PLOS ONE

Dear Dr. Morvinski,

Thank you for submitting your manuscript to PLOS ONE. After careful consideration, we feel that it has merit but does not fully meet PLOS ONE's publication criteria as it currently stands. Therefore, we invite you to submit a revised version of the manuscript that addresses the points raised during the review process.

I received two reviews from experts in the field. Both reviewers note major issues with your manuscript that preclude publication. Reviewer 2 is more positive than Reviewer 1, who argues for rejection. If you feel you can adequately address the reviewers' concerns, then I will allow you to submit a revised manuscript. If you decide to revise the paper, then I will send it back to the original reviewers. Note that the reviewers raised some serious concerns about the manuscript. Your manuscript would need to be extensively improved in order to convince the reviewers that your conclusions are supported by the data.

Please submit your revised manuscript by Jun 06 2022 11:59PM. If you will need more time than this to complete your revisions, please reply to this message or contact the journal office at plosone@plos.org. When you're ready to submit your revision, log on to https://www.editorialmanager.com/pone/ and select the 'Submissions Needing Revision' folder to locate your manuscript file.

Please include the following items when submitting your revised manuscript:

- *A rebuttal letter that responds to each point raised by the academic editor and reviewer(s). You should upload this letter as a separate file labeled 'Response to Reviewers'.*
- *A marked-up copy of your manuscript that highlights changes made to the original version. You should upload this as a separate file labeled 'Revised Manuscript with Track Changes'.*
- *An unmarked version of your revised paper without tracked changes. You should upload this as a separate file labeled 'Manuscript'.*

If you would like to make changes to your financial disclosure, please include your updated statement in your cover letter. Guidelines for resubmitting your figure files are available below the reviewer comments at the end of this letter.

If applicable, we recommend that you deposit your laboratory protocols in protocols.io to enhance the reproducibility of your results. Protocols.io assigns your protocol its own identifier (DOI) so that it can be cited independently in the future. For instructions see: https://journals.plos.org/plosone/s/submission-guidelines#loc-laboratory-protocols. Additionally, PLOS ONE offers an option for publishing peer-reviewed Lab Protocol articles, which describe protocols hosted on protocols.io. Read more information on sharing protocols at https://plos.org/protocols?utm_medium=editorial-

email&utm_source=authorletters&utm_campaign=protocols.

We look forward to receiving your revised manuscript.

Kind regards,

Darrell A. Worthy, Ph.D
Academic Editor
PLOS ONE

Dear Professor Worthy,

We thank you and the review team for taking the time to read our paper and provide constructive feedback; we are grateful for the opportunity to revise our manuscript for further consideration by the PLOS ONE journal. Following the guidelines provided by you and the two reviewers, we have revised the original version substantially. Thanks to the feedback, we think the quality of the manuscript has considerably improved and we believe that we now have a more complete paper that will appeal to PLOS ONE readers. We hope that the revised paper meets the expectations of the review team. Below, we detail the major changes we have made to the paper:

- Following the suggestion of Reviewer 2, we have heavily revised the front end and the general discussion of the paper. We now discuss the existing literature more thoroughly, elaborate on the theoretical basis of our predictions, and explain the proposed mechanism more carefully. In the general discussion, we further discuss theoretical contributions of the results.
- Given that the Kickstarter results raised major concerns by both reviewers, and the fact that the JGive data is a much richer dataset than the Kickstarter dataset, we did not include the Kickstarter study in the revised manuscript.
- In response to Reviewer 2, we present results on the likelihood of giving in results section of each study.
- We now publicly share the data, analysis scripts, and survey materials for studies 2 and 3.

In addition, we now include an Ethics Statement in the manuscript (p. 12) that is in compliance with the journal’s guidelines.

Below, we present a point-by-point response to each comment provided by the two reviewers. Per the journal’s requirements, we have highlighted major changes in the manuscript in blue text.

Thank you for the opportunity to revise this paper.

Sincerely,
The Authors

Journal Requirements:

When submitting your revision, we need you to address these additional requirements.

1. Please ensure that your manuscript meets PLOS ONE's style requirements, including those for file naming. The PLOS ONE style templates can be found at

https://journals.plos.org/plosone/s/file?id=wjVg/PLOSOne_formatting_sample_main_body.pdf and

https://journals.plos.org/plosone/s/file?id=ba62/PLOSOne_formatting_sample_title_authors_affiliations.pdf

Response: The manuscript style and formatting were adjusted accordingly.

2. PLOS ONE has specific requirements for studies using personal data from third-party sources, including social media, blogs, other internet sources, and phone companies (https://journals.plos.org/plosone/s/submission-guidelines#loc-personal-data-from-third-party-sources). These requirements include confirming data are collected and used in accordance with the company or website's Terms and Conditions, obtaining appropriate ethics or data protection body review, and ensuring appropriate consent from individuals whose data are used in research. In this case, please ensure that your Ethics statement is in compliance with guidelines, and that you have complied with the company's (i.e., Kickstarter and JGive's) Terms and Conditions, with appropriate permissions.

Response: Our Ethics Statement (p. 12) now specifies that we are in compliance with these requirements. We have included this statement below for your convenience:

All studies were approved (in writing) by the Human Research Protection Program of the University of California, San Diego (IRB number: 130572XX). Study 1 used data from the field and Studies 2 and 3 carried out at University of California, San Diego. Participants in Studies 2 and 3 were recruited from the Rady School of Management behavioral lab's subject pool and were undergraduate students who take part in laboratory studies for course credit. All participants in these studies provided written consent to participate. Study 1 data was provided directly by JGive with their permission to publish research findings and remained within their terms and conditions. Data, analysis scripts, and survey materials for Studies 2 and 3 are available on Open Science Framework (OSF) at https://osf.io/rb8s2/?view_only=80cf75c84fe940cf8860b6cb22633dde. Study 1 data is proprietary and cannot be shared publicly.

As explained below (see 5.1), we no longer present the Kickstarter study in the manuscript.

3. In your Data Availability statement, you have not specified where the minimal data set underlying the results described in your manuscript can be found. PLOS defines a

study's minimal data set as the underlying data used to reach the conclusions drawn in the manuscript and any additional data required to replicate the reported study findings in their entirety. All PLOS journals require that the minimal data set be made fully available. For more information about our data policy, please see http://journals.plos.org/plosone/s/data-availability.

Upon re-submitting your revised manuscript, please upload your study's minimal underlying data set as either Supporting Information files or to a stable, public repository and include the relevant URLs, DOIs, or accession numbers within your revised cover letter. For a list of acceptable repositories, please see http://journals.plos.org/plosone/s/data-availability#loc-recommended-repositories. Any potentially identifying patient information must be fully anonymized.

Important: If there are ethical or legal restrictions to sharing your data publicly, please explain these restrictions in detail. Please see our guidelines for more information on what we consider unacceptable restrictions to publicly sharing data: http://journals.plos.org/plosone/s/data-availability#loc-unacceptable-data-access-restrictions. Note that it is not acceptable for the authors to be the sole named individuals responsible for ensuring data access.

We will update your Data Availability statement to reflect the information you provide in your cover letter.

Response: Please use the following Data Availability statement:

Data Availability: Study 1 data was provided directly by JGive with their permission to publish research findings and remained within their terms and conditions. Data, analysis scripts, and survey materials for Studies 2 and 3 are available on Open Science Framework (OSF) at https://osf.io/rb8s2/?view_only=80cf75c84fe940cf8860b6cb22633dde. Study 1 data is proprietary and cannot be shared publicly.

4. We note that you have stated that you will provide repository information for your data at acceptance. Should your manuscript be accepted for publication, we will hold it until you provide the relevant accession numbers or DOIs necessary to access your data. If you wish to make changes to your Data Availability statement, please describe these changes in your cover letter and we will update your Data Availability statement to reflect the information you provide.

Response: As indicated in our previous response, a data repository is available here: https://osf.io/rb8s2/?view_only=80cf75c84fe940cf8860b6cb22633dde

A DOI will be provided at acceptance.

5. Please include your full ethics statement in the 'Methods' section of your manuscript file. In your statement, please include the full name of the IRB or ethics committee who

approved or waived your study, as well as whether or not you obtained informed written or verbal consent. If consent was waived for your study, please include this information in your statement as well.

Response: As mentioned, a full ethics statement including this information has now been added to the main text (p. 12).

6. Please include a caption for figure 2.

Response: A caption has now been added to this figure.

7. Please include captions for your Supporting Information files at the end of your manuscript, and update any in-text citations to match accordingly. Please see our Supporting Information guidelines for more information: http://journals.plos.org/plosone/s/supporting-information.

Response: Captions for Supporting Information files have now been added at the end of the manuscript (p. 31). DOI's will be provided at acceptance.

Thank you for the opportunity to revise this manuscript, we sincerely appreciate it.

Reviewers' comments:

Reviewer's Responses to Questions

Comments to the Author

1. Is the manuscript technically sound, and do the data support the conclusions?

The manuscript must describe a technically sound piece of scientific research with data that supports the conclusions. Experiments must have been conducted rigorously, with appropriate controls, replication, and sample sizes. The conclusions must be drawn appropriately based on the data presented.

Reviewer #1: No

Reviewer #2: Partly

---

2. Has the statistical analysis been performed appropriately and rigorously?

Reviewer #1: No

Reviewer #2: Yes

---

3. Have the authors made all data underlying the findings in their manuscript fully available?

The [PLOS Data policy](#) requires authors to make all data underlying the findings described in their manuscript fully available without restriction, with rare exception (please refer to the Data Availability Statement in the manuscript PDF file). The data should be provided as part of the manuscript or its supporting information, or deposited to a public repository. For example, in addition to summary statistics, the data points behind means, medians and variance measures should be available. If there are restrictions on publicly sharing data—e.g. participant privacy or use of data from a third party—those must be specified.

Reviewer #1: No

Reviewer #2: No

---

4. Is the manuscript presented in an intelligible fashion and written in standard English?

PLOS ONE does not copyedit accepted manuscripts, so the language in submitted articles must be clear, correct, and unambiguous. Any typographical or grammatical errors should be corrected at revision, so please note any specific errors here.

Reviewer #1: Yes

Reviewer #2: Yes

---

5. Review Comments to the Author

Please use the space provided to explain your answers to the questions above. You may also include additional comments for the author, including concerns about dual publication, research ethics, or publication ethics. (Please upload your review as an attachment if it exceeds 20,000 characters)

Reviewer #1:

1. Study 1a: The authors use snapshot data from different campaigns. I do not understand how the authors arrive at their conclusions but maybe I do not understand the structure of the data correctly. Do the authors have the information about the last donation? Then, one could analyze the size of the last recorded donation conditional on the completion rate, number of previous donors, and average donation of previous

donors. However, I think that the authors do not look at the incremental but at the average donation. In such a case the results are a kind of tautology. Think of two identical campaigns, A and B, with the same goal and exactly the same completion rate. If B has received more donations then, necessarily, the average donation in B must be lower. If this is a case, I do not see any value in presenting this data.

Response: You are correct that in Study 1a we look at the average donation of each project, rather than each incremental donation. Unfortunately, individual donations were not captured in this dataset. Your major concern about this data, together with the concerns raised by the other reviewer led us to decide not to present this study. We believe that the results from the Kickstarter data do not offer new insights other than a conceptual replication of Study 1b. Given that the JGive dataset is much richer than the Kickstarter dataset as it includes all individual donations, we believe that the results of the JGive analyses provide sufficient evidence from the field for our hypothesis.

2. Minor: Table 2: N is missing

Response: Thank you for pointing out this oversight, the N has now been included in the table, as well as BIC (for model comparison).

3. *Study 1b seems to be doing what I suggested above, however, I am not sure that the model is sufficiently taking care of the dynamics of the data. I wonder whether campaigns with many donors that result in many observations in the data, receive a too high weight, and whether this affects the coefficients. Does using weights [1/N] change the regression results? Actually, the authors have sufficient data to run separate (between projects) regressions, say using the incremental new donation amount after 70% of the goal has been reached (80%, 90%, etc). Then one could regress the new donation on the number of previous donations and the amount needed for completion. In the original regression, and the given setting, I am also worried about the correlation between the number of donors and the percentage of goal reached. Within one project there must be a very high correlation such that I am not sure how to interpret the coefficients. This would be taken care of in the regression proposed above.*

Response: It is our understanding that weighted least squares regression is most suitable when homoscedasticity assumption of constant variance in the error terms is violated. We were not sure whether this was your concern and whether weights of 1/N would necessarily alleviate it (but see below). Moreover, the over representation issue is more relevant in the former Study 1a, where we did not have individual donation information. If the concern is that some unobserved campaign characteristics that are unique to large campaigns may drive the results, we note that all our regressions include campaign random effects to account for such unobserved campaign-level variables. In fact, these are nested effects within charity random effects to account for the fact that a charity may run multiple campaigns over the investigated time frame. Note, having many donors in a campaign, holding other factors constant, is, in fact part of the effect we are trying to measure.

As you suggested, we present below regressions using subsets of the data by decile of goal progress for projects with at least 40% of the goal reached (40% is the predicted inflection point, see Fig 1). As you can see in Table 1 below, all deciles revealed a negative effect of number of donors on the donation amount. We noticed however, that some coefficients did not reach significance level (although they were directional) and we suspected that observations of very large campaigns that are included in some deciles but not in others, may behave slightly differently as you suggested. When we reran the analysis, this time excluding 131 campaigns with a fundraising goal of one million NIS or more, the coefficients of all deciles became significant (Table 2). Finally, since the excluded large campaigns had significantly more donors (Mean = 64.4) than the rest of the campaigns (Mean = 11.4), the latter result addresses the concern that the study results are due to campaigns with many donors/observations receiving too much weight.

We hope that these results, together with the controlled studies, helped convincing you that our effect is real.

Table 1. Deciles of Previous Goal Covered Regressions

	Dependent variable: log(donation amount)					
	Decile of previous goal covered:					
	40-50%	50-60%	60-70%	70-80%	80-90%	90-100%
Intercept	5.224***	5.233***	5.324***	5.504***	5.241***	5.083***
	(0.216)	(0.260)	(0.221)	(0.279)	(0.319)	(0.166)
Number of donors	-0.182	-0.021	-0.346**	-0.406**	-0.251	-0.157**
	(0.152)	(0.200)	(0.172)	(0.168)	(0.176)	(0.063)
Goal amount	0.935	0.823	1.626**	2.381**	1.821*	1.381***
	(0.771)	(0.926)	(0.777)	(0.949)	(1.082)	(0.363)
Has matching	0.154	0.130	0.400**	0.149	0.103	0.272
	(0.144)	(0.165)	(0.175)	(0.175)	(0.209)	(0.279)
Last donation	2.542***	2.417***	1.797***	1.712***	1.389***	0.862***
	(0.317)	(0.257)	(0.161)	(0.151)	(0.099)	(0.078)
Charity random effects	Yes	Yes	Yes	Yes	Yes	Yes
Campaign random effects	Yes	Yes	Yes	Yes	Yes	Yes
Observations	2,978	2,064	1,773	1,589	1,398	1,220
Bayesian Inf. Crit.	8,022.677	5,523.590	4,577.296	4,111.233	3,482.042	3,280.096

Note: *p<0.1; **p<0.05; ***p<0.01
Standard errors are presented in parentheses below parameter estimates.

Table 2. Deciles of Previous Goal Covered Regressions (Goal < 1M)

	Dependent variable: log(donation amount)					
	Decile of previous goal covered:					
	40-50%	50-60%	60-70%	70-80%	80-90%	90-100%
Intercept	5.993***	7.200***	6.587***	8.412***	5.916***	5.392***
	(0.498)	(0.668)	(0.683)	(0.843)	(0.652)	(0.415)
Number of donors	-0.633**	-1.717***	-0.957***	-1.586***	-0.519*	-0.206**
	(0.292)	(0.468)	(0.333)	(0.384)	(0.310)	(0.099)
Goal amount	3.744**	8.742***	6.026***	12.141***	4.086*	2.362*
	(1.763)	(2.445)	(2.313)	(2.813)	(2.207)	(1.267)
Has matching	0.130	0.069	0.368**	0.116	0.112	0.268
	(0.140)	(0.157)	(0.169)	(0.162)	(0.213)	(0.286)
Last donation	2.554***	2.417***	1.800***	1.712***	1.397***	0.863***
	(0.314)	(0.258)	(0.163)	(0.153)	(0.096)	(0.077)
Charity random effects	Yes	Yes	Yes	Yes	Yes	Yes
Campaign random effects	Yes	Yes	Yes	Yes	Yes	Yes
Observations	2,380	1,665	1,358	1,146	1,020	872
Bayesian Inf. Crit.	6,449.141	4,541.900	3,579.508	3,028.854	2,504.141	2,337.991

Note: *p<0.1; **p<0.05; ***p<0.01

Standard errors are presented in parentheses below parameter estimates.

4. Study 2: I think that the authors are overlooking a standard public good problem for which we know that with increasing N the average contribution is declining. The other issue is that of pivotality. If there is 86% of the goal reached and only 23 individuals donated previously that it is more likely that I am pivotal to finish the project while with the same amount reached by almost 2,000 previous donors, my donation is really negligible. (Strangely, there seem to be no information about the goal amount, but the goal amount must be expected to be larger with almost past 2,000 donations than with 23 past donations).

Response: We appreciate this comment and will first address the predictions one can make based on existing literature, followed by the topic of pivotality. Recent empirical work on the effect of group-size on contribution to public goods reveals mixed results. In fact, several recent publications showed that group-size has a positive effect on cooperation (Barcelo and Capraro, 2015; Diederich, Goeschl, and Waichman, 2016; Pereda, Capraro, and Sánchez, 2019). Furthermore, as we note in the manuscript (p. 5), research in psychology on descriptive norms indicates that the number or percentage of individuals engaging in a behavior increases, others will follow that behavior (Cialdini, Kallgren, & Reno, 1991; Cialdini, Reno, & Kallgren, 1990; Kallgren,

Reno, & Cialdini, 2000). Following your comment, however, we added discussion about the public goods problem to the introduction of the manuscript (p. 6).

Regarding the pivotality of the donation, we agree with your logic but offer and find a slightly broader interpretation. We believe that pivotality is another way to think of perceived progress and therefore this explanation is similar to ours. Indeed, we show that when learning that few (vs. many) people have donated, participants perceived their contribution to have a larger impact on helping the campaign reaching its goal, or as you described it, they felt that their contribution is more pivotal. We therefore don't see pivotality as an issue but rather as a different way to describe the results. Further, we note that pivotality is largest when one can complete the goal. While this is not an issue in the lab studies because the goal was unknown, larger final donations could potentially drive the results from the field. However, our field data results remain the same even when controlling for whether a donation was the last contribution to complete the goal (see Table 2 in the manuscript, p. 15).

Finally, we also considered the notion (and potential explanation) of participants making inferences about the average donation and then donating a similar amount. While we did not measure this inference directly in Study 2, we did in Study 3, and found that it did not drive our results.

5. Procedures: I am very worried about the deception in the material that the authors present to the subjects: where did they take the numbers 23/1923/86%/14% from? Similarly, were the participants aware of the probability of their decisions being implemented? I consider the probability of 1/571 to be really negligible for individual decisions. The authors should have rather chosen fixed probability of 1/10 or 1/20.

Response: The number of donors and goal progress used in Studies 2 and 3 were taken from pretests indicating that participants perceived 1923 as more donors than 23 (1 = a very small number, 7 = a very large number) and 86% as a greater goal completion rate than 14% (1 = very far, 7 = very close). The exact numbers are less important than whether there is a perceived difference in the size of these figures. In reality, many online fundraising campaigns do not "take-off" and the average number of donors is relatively small. For example, in the 2018 Giving Tuesday drives, the average number of donors per campaign was 21.8 (www.classy.org/giving-tuesday) and in 2016, the average number of donors per campaign in GoFundMe, the world's largest social fundraising platform, was 12.5 (https://pages.gofundme.com/giving-report-2016; In 2017, GoFundMe stopped sharing this information).

Regarding the probability of participants' decisions being implemented, participants were indeed not given this information. Incentivizing decisions on a probabilistic basis is a widely implemented strategy in behavioral science and many studies exclude information about the winning probabilities (e.g., Munichor & Steinhart 2015; Erlandsson et al. 2016, Perez, Steinhart, Grinstein, & Morren 2022). In fact, one review found that across studies, paying a subset of participants (versus all participants) produced little

change in results (Charness, Gneezy, & Halladay 2016). Because our measure of giving was a one-shot decision that was not effort- or skill-dependent, we believe that participants would behave as if their decision would be implemented regardless of the probability of implementation. We note that all participants received course credit for their participation and did not expect an additional payment when showing up at the lab. Even if we used a fixed known probability of the decision being implemented, which could alter participants' belief about their winning odds, this would only affect their motivation to give (i.e., main effect). That is, we have no reason to believe that changing participants' perceived probability to win the lottery should differentially affect their contribution between the few and many donors conditions.

6. Study 3 Introduction: I am confused when the authors talk about study 2 and 3 that "participants viewed … only accumulated progress." Was Study 3 not aiming at changing this?

Response: Our apologies for the typo, we have now deleted 'Study 3' from this sentence.

7. My remarks to the procedures are the same as those to study 2.

Response: We hope that our responses above have helped to address your concerns about Study 3.

8. Do the authors correct for multiple hypothesis testing?

Response: A post-hoc Tukey test did not change the results as they remained statistically significant. We now discuss this test in the results section of Study 3. We also note that in Study 3, our mediation analyses included all potential explanations simultaneously, so the appropriate p-value correction is built into the test procedure.

9. Implications:
I am very worried that the policy implications which the authors propose are not sufficiently backed up by the studies conducted. If the authors suggest that charities might split a large goal into smaller goals that could be achieved in smaller groups, why do not test this directly.

Response: We appreciate you pushing us to further demonstrate policy implications. However, suggestions of policy implications that are based on results from laboratory studies could always be criticized for lack of realism. We believe that testing the above policy implication in an additional field study would go beyond the scope of the current investigation. To alleviate your concern, in the new manuscript we also state that more

studies should directly test this strategy in real fundraising campaigns. If this suggestion still raises this concern, we will be happy to remove it.

10. Overall, I am worried that the findings are quite trivial and in line with basis economic models of public good contributions and related to pivotality. Of course, one can test simple theory but the authors should spell it out at the beginning. If the results are not with the simple theory then it would be good to search for other—behavioral—explanations. I suggest to drop Study 1a completely and move study 1b to the Appendix.

Response: As we note in our response to point 4, while our results are consistent with some economic models of public goods contributions, they run counter to recent public goods findings as well as to a large body of work on descriptive social norms. That is, while some public goods findings suggest that individuals will give less when others give more, other findings suggest the opposite. Moreover, social norm explanations suggest that individuals will give more when others give more. Given strong evidence that people largely conform to the behavior of others (e.g., social norms), we believe that our findings are not obvious.

You also mentioned that our results are in line with work on pivotality. As mentioned above, we don't see pivotality as an issue but rather as another contributor to the results. Note, however that we control for the 'completion effect' which is essentially pivotality as you describe it and still find the reported pattern of results. Moreover, our work generalizes beyond this by examining donations at late stages of campaigns where it may not be clear or plausible to donors that their donation will be the one to finish the project. Furthermore, to our knowledge, research on pivotality has not explored the joint impact of the percentage of the goal reached and number of previous donors, as we do in this research.

11. In addition, there are some papers on the completion effect in crowdfunding campaigns that the authors should include in their paper:
Argo, Nichole, et al. "The completion effect in charitable crowdfunding." Journal of Economic Behavior & Organization 172 (2020): 17-32.
Cox, J., Tosatto, J. and Nguyen, T. (2022), "For love or money? The effect of deadline proximity on completion contributions in online crowdfunding", International Journal of Entrepreneurial Behavior & Research, Vol. ahead-of-print No. ahead-of-print. https://doi.org/10.1108/IJEBR-04-2021-0317
I believe there are more.
In top, the dynamics of crowdfunding campaigns are studied in several papers by Cason and Zubrickas.

Response: Thank you for the recommendation, we have now cited these papers in the manuscript (p. 8).

Reviewer #2: Dear authors,

I am pleased to review your very interesting article about the interaction between number of donors and reaching funding goal on average donation amount. I think you are studying a very interesting, counterintuitive effect that might help us to understand the boundary conditions giving. Also, I highly value the overall study design, combining secondary, longitudinal data with experiments, as well as replicating findings in a different context (Study 1a and Study 1b). I am happy to provide suggestions to improve the manuscript.

Response: Thank you for the positive feedback, we appreciate it!

1) Data and syntax availability. Given recent open science practices, I would have expected to have access to data and syntax of the work. I understand that there will be an OSF repository after acceptance, and that some data (Study 1b) is proprietary. Still, as a reviewer, I would appreciate if I could receive a (anonymous) link to the data.

Response: Your request was very well taken, and we now made the data, analysis scripts, and survey materials for studies 2 and 3 publicly available. They are available here: https://osf.io/rb8s2/?view_only=80cf75c84fe940cf8860b6cb22633dde

As you noted, the JGive data cannot be shared due to propriety reasons and the Kickstarter analyses was not shared because as you will see below, following the review team aggregated feedback, we decided not to present Study 1a. Regardless, Kickstarter data is available from the following website: https://webrobots.io/kickstarter-datasets.

2) Introducing the scope of the project. The introduction might make clearer, at the beginning, why the two variables under study (and their interaction effect) are so important. Instead of presenting it as a possible counterintuitive effect, I would like to see some more urgency and rationale. Do you have empirical examples that hint at this effect? (so an interesting empirical puzzle, as an anomaly)? Or is the current literature missing/lacking explanations for a certain phenomenon. I found the third paragraph (starting with however) particularly confusing. The mechanism of the hypothesis remains unclear, and I also noticed that you write about the likelihood of giving, whereas you use the log of the average donation amount in the studies.

Response: Thank you for this suggestion. Following your feedback, we completely rewrote the introduction section. We now discuss the existing literature more thoroughly, elaborate on the different predictions of these theories, and explain the proposed mechanism more carefully. We hope that you will find the new introduction to your liking. Finally, we note that both studies 2 and 3 also analyze the probability of giving

and find similar results, but for brevity, we previously presented these results in the supplementary materials. We now present these analyses in the results section of each study, in the main text.

3) Theoretical grounding. While the empirical approach is sound, and methods are described in a clear matter, I think the manuscript can improve in terms of theoretical grounding. I think the main point is that I could not understand whether it is a deductive or inductive paper. On one hand, it is deductive since you propose a central, interaction hypothesis in the introduction. On the other hand, the paper lacks a conceptual model and hypotheses (except the brief part in the introduction). But, even when it is more inductive, I would have expected more grounding in prosocial and fundraising literature. I would suggest to provide more argumentation in the introductory parts of the studies. Last, I think that the discussion could be improved by elaborating on the theoretical implications, and possible alternative explanations (e.g. I had to think about Bandura's collective efficacy as a potential mechanism). The contributions to the prosocial literature remain unclear.

Response: We agree that the theoretical grounding of the paper should be clearer and more detailed. We have now expanded the introduction, discussing how our work draws on the focus theory of normative conduct (Cialdini, Kallgren, & Reno, 1991; Cialdini, Reno, & Kallgren, 1990; Kallgren, Reno, & Cialdini, 2000). We have also provided a more thorough review of the social norms and fundraising literatures. Furthermore, we have now extended and clarified discussion of the potential mechanisms underlying the hypothesized effect. We hope that these changes now more effectively motivate our research question and hypotheses.

4) Methods. First, I was wondering whether the Kickstarter case fits your RQ and scope. I have participated in several Kickstarter projects, and while there might be some prosocial motivations, it is often more of an investment (with the promise of a product/service) than a donation. Hence, the empirical context of Kickstarter is somehow disconnected from the RQ, and hence I am not surprised that the findings between Study 1a and 1B differ. Second, I noticed you worked with samples of students for your experiments. It might be possible to use students for experiments, but it is common practice in Psychology to explain why students would be a suitable population to study your RQ. Third, while I actually liked that the experiment included a real cause (increasing the psychological realism), I was surprised by the high percentage of respondents giving money. This might be an important limitation: does the empirical approach (experiment) somehow put pressure to give (a small amount of) money to the cause, making the behavior less realistic?

Response: While several publications investigate prosocial behavior in the context of Kickstarter projects, and even demonstrate that contributors on Kickstarter have prosocial motivations to help creators reach their funding goals (e.g., Dai & Zhang 2019), we agree that whether reward-based crowdfunding platforms such as Kickstarter are a good context for studying prosocial behavior may still be questionable to many

scholars. We therefore take your comment to heart. In addition, the validity of the Kickstarter data was a major concern of the other reviewer. Given that the JGive data is much richer than the Kickstarter data as it includes all individual donations, we believe that the results of the JGive analyses provide sufficient evidence from the field for our effect. Thus, we decided not to present the Kickstarter study in the revised manuscript.

Using student samples to study donation decisions is a common practice in many published papers as students often create, manage, and donate to fundraisers (e.g., Van Baaren et al., 2004, Frey & Meier, 2004; Martin & Randal, 2009 Siugmin et al., 2020). Since this was not a new practice, we did not think that explaining why students would be a suitable population to study our research question was necessary. Still, following your comment, we added language in the general discussion section highlighting the potential limitation of this approach (p. 30).

We agree that the donation rate in the lab experiments may seem higher than in normal giving contexts. However, two reasons may partly explain this result. First, students were offered to donate from an additional bonus which was on top of the expected course credit they received for their participation. Second, the donation decision was not an all-or-nothing decision but instead students could donate a portion of their $20 bonus. Indeed, the average donation was significantly smaller than $20 indicating that most participants did not donate the entire amount but instead kept some portion of the bonus for themselves. We note that the reported percentage of respondents giving money includes any donation greater than zero. Given that the findings from these experiments are supported by the data on real campaigns in the JGive data, we believe that the lab results are generalizable. Still, we now discuss this concern as a potential limitation (p. 30). Thank you for pointing out these important issues.

I hope my suggestions and questions help the authors to further improve their manuscript.

Thank you very much for your insightful feedback and support.
