# Supplementary material for: Social information decreases giving in late-stage fundraising campaigns
Source: PLoS One. 2022 Dec 1;17(12):e0278391. doi: 10.1371/journal.pone.0278391 (PMC9714697; doi:10.1371/journal.pone.0278391)
Supplement: Attachment — Submitted filename: Giving Plos one Response Letter R2.docx [file pone.0278391.s003.docx]

PONE-D-22-05379R1

Social Information Decreases Giving in Late-stage Fundraising Campaigns

PLOS ONE

Dear Dr. Morvinski,

Thank you for submitting your manuscript to PLOS ONE. After careful consideration, we feel that it has merit but does not fully meet PLOS ONE’s publication criteria as it currently stands. Therefore, we invite you to submit a revised version of the manuscript that addresses the points raised during the review process.
I sent your paper back to one of the original reviewers, and the reviewer still lists several concerns.  If you feel you can address these concerns then I invite you to submit a revision.  If the reviewer still has major concerns after the next round of review then I will likely reject the paper, so please give your best effort to adequately address the remaining concerns raised by the reviewer.

We look forward to receiving your revised manuscript.

Kind regards,

Darrell A. Worthy, Ph.D
Academic Editor
PLOS ONE

Dear Professor Worthy,

We thank you for inviting us to submit a revision. We are also grateful for the reviewer for taking the time to read the previous revision and share her/his feedback. As you will see in the new revision, we heavily revised the front end of the paper: We now better explain the theoretical reasoning, and as suggested by the reviewer incorporate the second potential mechanism into the theory, as well as separate the hypotheses for clarity. We also address additional requests by the reviewer, such as providing data documentation.

Thanks to the reviewer feedback, we believe that PLOS ONE readers will find the manuscript easier to follow as the comprehensive introduction nicely leads to the empirical evidence. We hope that you will also share this view.

Below, we present a point-by-point response to each comment provided by the reviewer. Per the journal’s requirements, we have highlighted major changes in the manuscript in blue text.

Thank you for the opportunity to revise this paper.

Sincerely,

The Authors

6. Review Comments to the Author

**Reviewer #2:** Thank you for the opportunity to review the revision of your interesting work. Overall, I applaud the authors for the amount of work that went into this revision. The authors really engage with the suggestoins and questions of the previous round. Still, I have some remaining concerns.

- I appreciate that the data is partly available. I miss documentation (e.g. metadata) to understand the dataset properly.

**Response**: Thank you for this suggestion. We now added a data dictionary file to each study explaining the dataset properties.

- While the authors has put a lot of work in rewriting the introduction, I still think that the rationale and argumentation is convoluted. I don’t understand the mechanism “Similarly, giving to a nearly completed campaign with few (versus many) donors may signal that one’s donation will make a greater marginal impact on goal progress, which consequently increases motivation to give”, since the gap to be bridged to the end goal is the same for campaigns with many and with few donors. Alternatively, could it not be that if there are only few donors (but the end goal is almost reached) that a potential donor estimates the average norm for a donation is high, setting a baseline for his/her own donation? Counterintuitively, you would expect that the likelihood of giving would go down, because the threshold of giving is higher due to this descriptive norm.

**Response**: Thank you for this comment, you raised a few important points. First, following your comment above we realized that we did not explain clearly enough why a donation that is part of few (vs. many) contributions is perceived to have a greater marginal impact on the goal. You are correct that in reality, the gap to be bridged to the end goal is the same for campaigns with many and with few donors. However, it is the *perceived* marginal impact (or share of responsibility) that each donation has on the goal progress that changes, particularly towards the end-goal. The perceived impact stems from the fact that in the few (vs many) condition, on average each individual donor is accounted for a greater percentage of the overall goal progress. Thus, a donor in the few condition may in turn reason that their subsequent donation will lead to marginally greater goal progress (i.e., have a larger impact on goal progress) compared to a donor in the many condition. Simply put, holding donation amount constant, being the thousand donor feels less impactful than being the tenth donor even if both donations make the same progress because the responsibly for the progress level reached following the donation is shared with many more donors. We do not expect this increase in the perceived share of responsibility when the campaign goal is far as each donation reduces a very small proportion of the *remaining* distance to the goal. We have now sought to better clarify this reasoning in the manuscript (pp. 11). This also explains the interaction between goal progress and number of donors observed in Study 1. Following your comment, we rewrote large parts of both the introduction and the theoretical development and explain this concept more clearly.

Second, we agree that in the few donors condition, people may estimate that the average donation is higher and thus give larger amounts. Following your recommendation, we now formally introduce this mechanism as potentially contributing to this effect, albeit to explain donation size, but not donation likelihood. At least in the context of our studies, we found no evidence that a higher perceived average donation drove the results

Finally, you brought up a very important point which we did not think about before. Indeed, a larger perceived average donation amount may set a higher threshold for giving which in turn may decrease rather than increase motivation to give. Although we observed a positive effect of the few donors condition on both donation amount and the likelihood of giving (Studies 2 and 3), we believe that it is important to discuss this potential mechanism, which we now do (pp. 5). Thank you for pointing this out.

- The new paragraph on theoretical and practical contributions is very generic, and should be more precise in how this paper contributes, and why this contribution is so urgent to understand.

**Response**: Thank you for this suggestion. We agree that we should have been clearer on the practical and theoretical contributions of this work. As such, we have now separated this section into two paragraphs (pp. 5-6), where we first discuss how fundraisers are increasingly moving to the online space, and how, despite their important role in helping to solve global issues, most fundraising goals go unmet. Thus, our research has important practical implications for online fundraisers by highlighting how the provision of both goal proximity and social information can backfire. It also offers suggestions on how fundraisers can implement these findings to increase donations to their campaigns. Furthermore, we explain how our research extends the focus theory of normative conduct by highlighting a novel boomerang effect. Unlike most previous research on the backfiring of social norms, or “boomerang” effects (e.g., Allen, Eilert, & Peloza, 2018; Cialdini, 2003; Schultz, Nolan, Cialdini, Goldstein, & Griskevicius, 2007), we highlight situational conditions under which observing a less normative action (i.e., less people are willing to donate) can lead to more of that behavior, compared to when the action is more normative. Further, we illuminate a social component of goal-oriented motivation, whereby the behavior of others influences the degree to which perceived goal progress motivates goal-directed behavior. We hope that these edits clarify the contributions of this work.

- I appreciate the further elaboration of the theory section. However, I found the hypothesis development quite unclear and ungrounded. First, the argumentation for the specific hypotheses is not backed with literature. The paragraph starting with “In this research, we hypothesize that descriptive norm information about the number of donors to a fundraising campaign can also increase perceived progress towards goal completion…” on p. 9 does not refer to research. So, the reasoning for a higher motivation towards an end-goal is clear, and the reasoning for a positive effect of fewer previous donors on giving is clear, but the interaction between the two remains ungrounded. Second, I would split the argumentation for hypotheses 1 and 2.

**Response**: Previous research has indeed not explored the interaction between goal proximity and social information on giving, which is why we do not cite any such literature. This gap in the literature partly motivated us to study this interaction – we hypothesized and found that when presented simultaneously, this information can interact in ways that run counter to what prior work would predict.

As you also suggested, we now split the hypotheses for greater accuracy and clarity.

- The paragraphs about alternative mechanisms in the theory section confuses the reader. It might be better to move and integrate this in the discussion.

**Response**: Thank you for this suggestion. We have revised and streamlined this paragraph, but decided to keep its location for completeness and clarity, as well as to motivate the additional measures we employed in the studies to test for the role of these mechanisms.

- Thank you for further explaining the methodological details. I appreciate your decision to remove the Kickstarter study. Still, I question the ecological validity of the experiments after reading your explanation of the specific population and the incentive/bonus.

**Response**: We agree that laboratory experiments from non-random samples of the population may always raise concerns about ecological validity. Hence, there is a need for researchers to corroborate laboratory results with studies in more externally valid settings. In this case, we provide those results in Study 1, where we identify our hypothesized effect in the setting to which we are attempting to generalize—online fundraising campaigns. While Study 1 does not provide causal evidence, Studies 2 and 3 do. Thus, the studies complement each other. In sum, we feel that the convergent evidence presented across these multi-method studies with widely varying samples and designs lends support for our conclusions.

We are grateful for your insights and suggestions and find the revised paper much improved. Thanks again for the time spent improving this important research.
